# Fractal parameters and vascular networks: facts & artifacts

**DOI:** 10.1186/1742-4682-5-12

**Published:** 2008-07-17

**Authors:** Daniele Mancardi, Gianfranco Varetto, Enrico Bucci, Fabrizio Maniero, Caterina Guiot

**Affiliations:** 1 Department of Clinical and Biological Sciences, University of Torino, ASO San Luigi, Regione Gonzole, 10, 10043, Orbassano, Torino, Italy; 2 Bioindustry Park del Canavese, Colleretto Giacosa, Torino, Italy; 3 Department of Oncological Sciences and Division of Molecular Angiogenesis, Institute for Cancer Research and Treatment (IRCC), University of Torino Medical School, Strada Provinciale, I-10060 Candiolo,Turin, Italy; 4 Department of Neuroscience, University of Torino, C. so Raffaello, 30, 10125, Torino, Italy

## Abstract

**Background:**

Several fractal and non-fractal parameters have been considered for the quantitative assessment of the vascular architecture, using a variety of test specimens and of computational tools. The fractal parameters have the advantage of being scale invariant, i.e. to be independent of the magnification and resolution of the images to be investigated, making easier the comparison among different setups and experiments.

**Results:**

The success of several commercial and/or free codes in computing the fractal parameters has been tested on well known exact models. Based on such a preliminary study, we selected the code Frac-lac in order to analyze images obtained by visualizing the angiogenetic process occurring in chick Chorio Allontoic Membranes (CAM), assumed to be paradigmatic of a realistic 2D vascular network. Among the parameters investigated, the fractal dimension D_f _proved to be the most robust estimator for CAM vascular networks. Moreover, only D_f _was able to discriminate between effective and elusive increases in vascularization after drug-induced angiogenic stimulations on CAMs.

**Conclusion:**

The fractal dimension D_f _is likely to be the most promising tool for monitoring the effectiveness of anti-angiogenic therapies in various clinical contexts.

## Introduction

The concept of fractal dimension was first introduced by Hausdorff [1] as a generalization of the geometrical dimension, and subsequently developed by Kolmogorov & Tihomirov [2]. By introducing a scale ε, according to which the original length of a segment is partitioned, and counting the number N of self-similar parts resulting from the partitioning, the fractal dimension D is defined as:

(1) D = log_ε _N

**Figure 1:**
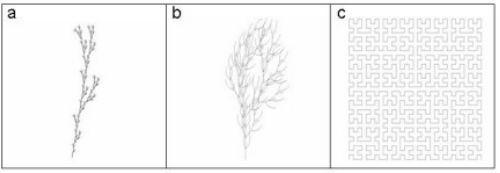
Images representing 3 different fractal networks: a) 'bush1', D_f _= 1,46; b) 'bush3', D_f _= 1,5, c) 'Hilbert', D_f _= 2.

When D is integer, it reduces to the current geometrical dimension (i.e, by partitioning in 3 equal parts the sides of a square, we obtain 2 = log_3 _(9), doing the same with a cube we obtain 3 = log_3 _(27), etc). However, also non-integer values of D are possible, corresponding to different 'recipes', e.g. by dividing a segment in 3 parts (ε = 3) and considering a new figure in which N = 4 segments form a cusp (such as in the Koch curve). Another example is the branching structure with N = 5 equal branches (as in the 'bush1' configuration, see Figure 1). Such plots produce a 'covering' of the plane which is intrinsically self-similar or 'scaling' invariant.

Although, in mathematical terms, a structure can be analyzed at an arbitrary high resolution, and D is actually defined as a 'limit', when the 'conceptual' structure is plotted as a 'real' image, the original scale-independence is lost. Considering the image of a self-similar structure, two intrinsic limits, i.e. the image resolution and its dimension, define the minimal (ε_m_) and maximal (ε_M_) values for the scale parameter ε. When the image of a fractal structure is considered, in order to apply Eq. (1), a practical approach for estimating D would be that of selecting a series of values for ε (ε_m _< ε < ε_M_), perform for each of them a tessellation of the image in boxes and compute the number N_b _of boxes containing the image structures (box-counting method, BCM), introduced by Mandelbrot [3]. For a detailed description of the method see Bunde & Havlin [4].

Results are clearly dependent on the procedure of tessellation and of evaluation of the linear regression. The quantification of such a procedure for different computational algorithms implemented in the most popular codes is the goal of the first part of our study.

In the real world, self-similarity becomes an even less well-defined concept. Accordingly, structures are generally defined as 'fractal-like', or by means of truncated fractals. First, real structures, although similar to segments, possess a 'thickness' (which can be disregarded provided some 'skeletonization' procedure is performed), which adds some 'noise' to the ideal fractal structure to which it resembles. Moreover, the minimal and maximal values within which the scaling behaviour is restricted (i.e. self-similarity is satisfied) can be further reduced by the structure itself. This point is of great importance for the biological systems, which have been recently investigated using scaling relationships by Brown and colleagues [5].

The rationale for an approach based on the assessment of fractal properties resides on previous studies on self-similar architectures observed in many biological structures, such as the bronchial tree [6] and the placental villous tree [7] and on the occurrence of scaling relationships (i.e., when two variables X and Y relate according to a given power law:

(2) **Y = Y_0_X^*p*^,**

As an example, Y can be the basal metabolic rate, X the body mass of living organisms and *p *is a non-integer, fixed value (*p *= 3/4 according to Kleiber [8]and West et al [9]). In another example Y is the basal metabolic rate, X the tumor mass and *p *a non-integer value changing according to the developmental phase of the tumor itself [10].

Such scaling properties are actually assumed to be originated by the microvascular structure, which is responsible for the delivery of nutrients to body cells. In principle, a generic branching system, whose purpose is to exchange nutrients at its endpoints, may satisfactorily develop without restrictive rules, such as self-similarity. However, optimalization (i.e. maximal delivery of nutrients at the endpoints) is reached imposing some constraints on the vessels length and diameters, in order to minimize energy expenditure for flow and metabolic purposes, which can be expressed as a power law relation between parent and daughter vessels' dimensions (see Zamir, [11]). As West pointed out [9], power exponents *p *are expected to be different in the pulsatile regimen (arteries, for which at each branching point, the area is preserved to minimize wave reflection) and in the microcirculation.

Finally, almost no geometrical constraints are expected for vascular networks induced by angiogenesis and elicited by various 'growth factors' produced by proliferating tissues, e.g. tumors. The characteristics of such vascular patterns are mainly due to the interplay with the host, and vascular growth is driven by local information about pressure, blood velocity, and by the presence of some randomness and noise. This point was emphasized by Sandau & Kurz [12,13], who suggested an extension of the concept of fractal dimension, called 'complexity', which best describes the case of non self-similar networks. Other parameters, related to the vascular network positional, topological and orientational orders were introduced by Guidolin et al [14].

Actually, the former considerations explain why Baish, Jain & Gazit [15] found that the in-vivo estimation of the fractal dimension of planar vascular networks in normal tissues and in four different tumor lines, implanted in the dorsal skinfold chamber of immuno-deficient mice ranges between the value of 2 for normal capillaries, 1.7 for arteries and veins and 1.88 for tumor vessels.

A similar estimation done on casts, derived from the application of other models proposed in the literature, gives values of 2 for the 'space-filling' growth model, 1.71 for the 'diffusion limited aggregation' model (simulating the arterio-venous system) and 1.90 for the 'invasion percolation' model (simulating the tumor neovasculature).

Due to the possible relevance of the fractal parameters for characterizing the neovascular tumor structures, which may be of interest for both diagnostic [16] and therapeutic [17] purposes, we wish here to investigate the vascular fractal dimension in the simplified model of the Chorio-Allontoic membranes (CAM) of chick eggs. A comparison with other parameters currently evaluated is performed. Moreover, since different expression of pro- and anti-angiogenic factors should elicit differences in the microvascular network development, we challenged the CAM with such compounds and evaluated their effects on the investigated parameters.

## Methodology

### Definition of the parameters

As previously stated, many geometrical forms in nature and, in particular, many vascular networks are thought to be 'fractal-like', i.e. they can be subdivided, up to a given scale, into 'self-similar' parts. The most popular (and potentially useful) parameters defined to describe fractals (from here on named 'fractal parameters') are the fractal dimension D_f_, which is the 'experimental' counterpart of D defined in Eq. (1), and the lacunarity L. Qualitatively, D_f _specifies how completely a fractal-like structure fills the space for decreasing scales, while the lacunarity assesses its texture, i.e. the distribution and size of the empty domains. The operative definitions given in our paper are the following. Images of the vascular network (of linear dimension Λ), obtained from any technique (microscopy, RMN, etc) are normally pre-processed in order to reduce their grey levels to a dichotomic (black/white) binary figure, or sometimes even skeletonised, i.e. the difference in diameter of the branches is neglected. Then a matrix of squares of side l = εΛ, with ε spanning in a given range (ε_m _< ε < ε_M_), is superposed on the binary image and the corresponding number N_b _of boxes containing at least one black 'pixel' of the image, is computed.

This procedure, using Eq. (2), with Y = N_b_, Y_0 _= 1 and X = ε, allows to define D_f _= p. In other words, D_f _is computed as the slope of the straight line Log N_b _= D_f _log ε in a log-log representation. If the variable under investigation is the variance over the square of the mean number of black points in the box of side l = εΛ, being σ the standard deviation and μ the mean, i.e. Y = (σ/μ)^2 ^and Y_0 _= 1 then, from Eq. (2) we can argue that the lacunarity of the image is L = *p*. By virtue of their definitions, both D_f _and L are expected to be scale invariant (**in a given range) **according to Eq.(2).

Among non-fractal parameters, the most diffused is the *Vascular density *V_d_, which is generally evaluated by estimating the fraction of the image area covered by vessels. Also the distribution of diameters and lengths of the vessels, as well as the number of generations in the tree-like networks, are often evaluated [18]. In order to monitor angiogenesis, the number of neo-formed vessels has been quantified by counting the number of network nodes per unit section, called *angiogenic index *or *forks density F*_*d *_[19].

### Validation of the parameters estimation methodology by applying various computational codes on 'exact models'

A few examples of 'exact models', i.e. '**fractal-like' **trees generated by dedicated SWs (e.g. the shareware 'treegenerator', ) were considered, some of which simulating the arterial tree (as the 'bush1' and 'bush 3' models, see Figure 1) and the capillary network (the so-called 'Hilbert' model, see Figure 1). Their D_f _is intrinsically defined by the constitutive constructive algorithm, i.e. the 'bush1' structure is obtained by dividing the initial segment into 5 sub-segments of length equal to 1/3 of the initial one, and replicating the above process. Therefore its fractal dimension is D_f _= ln5/ln3 = 1.465. Analogously, 'bush 3' was proved to have D_f _= 1.5 and the Hilbert network D_f _= 2. No 'exact' estimations for the lacunarity L of the same models are available. We will therefore simply check its scaling properties.

It is straightforward to observe that, for what concerns the vascular density V_d _and the number of forks F_d_, no scale-invariance is expected. Images of trees extended to different generations were generated, in order to check the ability of various programs, mainly working on the basis of the BCM, to estimate the correct D_f _and L values given by the exact calculations independently from the network extension. The code 'Winrhizo' was applied to simple fractal models to estimate F_d_.

The commercial codes compared in this paper are 'Fractalyse', (ThèMA, F), Whinrhizo' (Regent Instruments Inc.) and Image Pro Plus (Media Cybernetics), while FDSURFFT is a MATLAB^® ^routine and FracLac is a freely downloadable Plugin of ImageJ .

Table 1 summarizes the results for D_f _obtained by means of several codes. Data are missing for the cases in which the program was unable to give an estimation.

**Table 1:** evaluation of D_f _using different SW codes

	**D_f _exact**	**D_f _FDSURFFT**	**D_f _fractalyse**	**D_f _whinrhizo**	**D_f _ipp**	**D_f _frac_lac**
bush1_3	1,46	1,41	1,26	1,42	-	1,43
bush1_4	1,46	1,43	1,30	1,43	1,41	1,45
bush1_5	1,46	1,44	1,30	1,42	1,45	1,46
bush1_6	1,46	1,44	1,61	1,43	1,45	1,46
bush3_3	1,5	1,22	1,66	1,52	1,5	1,66
bush3_4	1,5	1,33	2,01	-	1,88	1,75
bush3_5	1,5	1,61	1,90	-	1,98	1,77
Hilbert5	2	-	2,07	1,67	1,05	1,73
Hilbert6	2	-	1,98	-	2	2,06
Hilbert7	2	-	1,98	-	2	2,06
Hilbert9	2	-	1,98	-	2	2,09

The lacunarity L was also evaluated for the same exact models using FracLac. After a direct verification that L changes very slightly with the number of generations (according to the expected scaling properties), we found (mean ± Standard Deviation): L = 0.374 ± 0.008 for 'bush 1', L = 0.67 ± 0.04 for 'bush 3' and L = 0.17 ± 0.02 for Hilbert.

To summarize, according to Tab 1, only FracLac could evaluate D_f _at best for all the exact models. Some of the programs, optimized for branching structures, such as FDSURFFT and Winrhizo, performed well for the 'bush' models but failed with 'Hilbert', while programs optimized for capillary structures, such as Image Pro Plus and Fractalyse, failed in estimating D_f _for branched structures. Moreover, FracLac can estimate lacunarity. As far as limitations are concerned, we conclude that some computer codes addressed to the evaluation of the fractal dimensions perform well only in limited contexts (for instance for tree-like and/or capillary-like structures), and it is therefore necessary to check the code performance before application.

## CAMs as models for 2d vascular trees

### Estimation of the parameters on the CAMs: scaling properties and robustness

**Figure 2:**
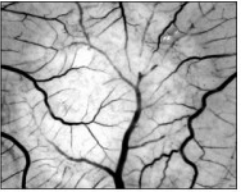
**Picture of chorio-allontoic membrane (CAM) of chick embryo**. The lighter round area represents the Watman paper disk (diameter = 6 mm). Magnification 10×.

The Chorio-Allontoic Membrane (CAM) in the fertilized hen egg starts to develop on the 5th day of incubation by the fusion of the chorion and the allantois [20]. It is completely formed on the 11th day and lies attached under the inner eggshell membrane (See Figure 2). Throughout CAM development, blood vessels and capillaries expand intensively [20,21]. The angiogenesis has been monitored using several parameters. For the chick embryo, Kirkner et al [22] showed that D_f _exhibits an almost linear dependence on the incubation time, from around 1.3 at day 6 up to 1.8 at days 13–14, followed by a decrease in the later stages of maturation. A similar pattern was shown by Parsons-Wingerter for the quail embryo [23]. Their results (obtained by using the MATLAB-supported VESGEN code) have been validated using the FracLac code on the same images.

Six eggs were incubated at 37°C in a humidified environment. After 72 hours eggs were oriented and windowed, 6–10 ml removed from the egg and embryo were visually checked for heart beat (vitality). At day 10 Watman paper discs (6 mm diameter) were exposed to UV, soaked in hydrocortisone (3 mg/ml in ethanol 100%), dried and placed on CAMs. Contaminated CAMs were excluded from the study. After treatments, membranes are fixed with 3.7% PAF in PBS and after 10 min CAMs were removed and placed into a Petri dish containing PAF. Discs were excised and imaging performed a JVC TK-C1380E color video camera (ImageProPlus 4.0 imaging software) connected to a stereomicroscope (model SZX9; Olympus). Pictures, taken at different magnifications, were processed with the dedicated software.

**Figure 3:**
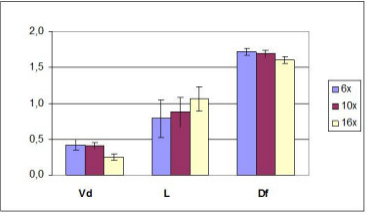
Comparison of the values of the parameters V_d _(vascular density), L (lacunarity) and D_f _(fractal dimension) from images of untreated CAMs taken at different magnifications.

At first the scaling properties were tested among three different magnifications (6×, 10× and 16×) for the parameters V_d_, L and D_f_, showing that both V_d _and D_f _vary significantly depending on the magnification only when it is larger than 10 (p < 0.05 ANOVA), while no test for L was reliable due to the huge data dispersion (see fig 3).

In conclusion, D_f _satisfies the scaling properties (as well as V_d_), provided images are taken at low and intermediate magnification (< 16 x).

In order to test the parameters robustness, i.e. suitability with the (sometimes unpredictable) variations in its operating environment, we applied the code FracLac on the six images (magnification 10×) to estimate D_f_, L and V_d_. Each evaluation of D_f _and L was performed averaging results from four computational procedures, which assumed as starting point for the box counting algorithm the 4 different corners of the image.

**Figure 4:**
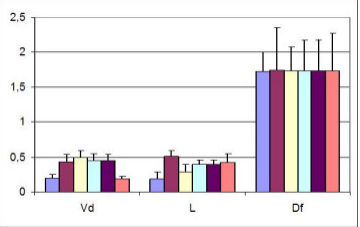
V_d_, L and D_f _evaluated on untreated CAMs.

Results are given in figure 4. It is apparent that D_f _is very consistently defined (no statistically difference, p < 0.05), and the final value is 1,733 ± 0,006. On the contrary, the result for L is affected by a large deviation (0,36 ± .0,11), producing a percentage error of about 50% and vanifying any further statistical analysis Also the non-fractal parameter V_d _shows a much larger, statistically significant variability, i.e. V_d _= 0,37 ± 0,14 (p < 0.05).

In conclusion, D_f _proved to be a very robust parameter, showing only very slight changes among the estimated values on different CAMs. Moreover, the value of D_f _estimated on CAMs confirms its characteristics of being an efficient distributive system.

### Estimation of the parameters on the CAMs: testing sensibility and specificity

In order to test whether the parameters are sensitive (i.e. can correctly identify a condition of enhanced vascularisation without false positives) and specific (i.e. can correctly identify a condition of apparent increase of vascularisation, without false negatives), we performed a drug response analysis. The main application of the studies reported in the literature was the investigation of CAMs response to various proangiogenic drugs (mainly VEGF and FGF) in terms of vascular architecture variation. For instance, when administered at concentrations between 1,25 and 2,5 μg, VEGF 165 induces increases in arterial density and in arterial diameter. Correspondingly, D_f _was shown to increase from 1,65 ± 0,01 to 1,69 ± 0,01 at its maximal dose [24,25].

Another commonly used proangiogenic compound is the Fibroblast Growth Factor 2 (FGF). Guidolin's group showed a significant increase in D_f _from 1,51 ± 0,01 of the control to 1,62 ± 0,04 after treatment at day 12 at the dose of 500 ng. On the contrary, L decreases from 0,288 ± 0,02 to 0,198 ± 0,02 [14]. The same group showed that, provided the angiogenetic process is antagonized with angiostatic factors, the fractal dimension of the vascular network reflects the observed decrease in branching patterns (about 10% from the starting value of 1.20) [14]. Other drugs proved to be far less effective in promoting angiogenesis. For instance Angiopoietin 1 (Ang), a ligand for the Tie2 receptor of endothelial cells, acts as a regulator of angiogenesis in several experimental models, although it is not clear whether it has a pro- or anti-angiogenic effect [26-31].

**Figure 5:**
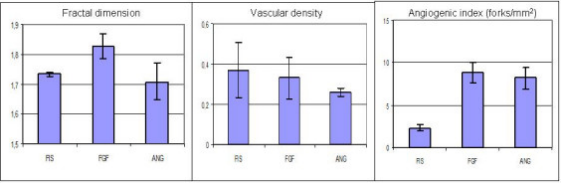
a, b, c: variations of D_f_, V_d _and F_d _according to different treatments (FGF, Ang).

On six CAM specimens, prepared according to the procedures already described above, the fractal dimension D_f _(using FracLac), the vascular density V_d _and the fork density F_d _(using Winrhizo) were computed after administration of FGF and Ang. Statistical analysis was performed using the Kruskal-Wallis nonparametric test. Following the administration of FGF and Ang, we obtained the following results (Figures 5a and 5b): D_f _for the control was (1.733 ± 0,006), while after administration of FGF increased to (1,826 ± 0,042, p < 0,05) and after administration of Ang was not statistically different versus the control (1,709 ± 0,061).

As far as the vascular density is concerned, in the control V_d _was (0,37 ± 0,14), after FGF was (0,33 ± 0,10) and after Ang was (0,26 ± 0,02), with no statistical difference among groups. Finally we examined the third index, i. e. the number of forks per mm^2^, F_d _finding that in the control it was (2,3 ± 0,4), after FGF administration became (8,9 ± 1,2) and after Ang (8,2 ± 1,3). The fork density increased significantly for both FGF and Ang treatment (see Figure 5c).

## Conclusion

Fractal parameters, such as the fractal dimension and lacunarity, have been widely used to investigate vascular systems, particularly those formed by neoangiogenesis. In this contribution we have at first validated several computational codes and discussed some of their possible weaknesses in the estimation of the fractal parameters. We then applied the code FracLac to CAM images. We found D_f _= 1,733 ± 0,006 at day 10, which is consistent with literature reports. We noted that the parameter D_f _is very robust, reproducible and reliable, in contrast with the other fractal (L) and non-fractal parameters (V_d _and F_d_) considered.

The most remarkable result of our analysis is that completely different conclusions can be drawn from the same set of data following drug treatments depending on the chosen parameters.

The density of forks F_d _showed a marked increase after administration of both Ang and FGF, suggesting that both drugs are equally effective in promoting branching. On the contrary, none of the drugs affected the vascular density V_d_, with a surprisingly discrepancy with respect to expectations from the literature [14,32].

On the same set of data D_f _resolved a finer discrimination between the pro-angiogenic effect of FGF compared to the more controversial regulatory effect of Ang. It proved therefore to be both sensitive for the angiogenic affect of FGF and specific for the ineffectiveness of Ang.

According to Stoeltzing[31]Ang possibly plays a role in a later phase of developmental angiogenesis, such as remodelling and maturation of vessel network. Such effect, although of great importance on a biological ground, is not expected to modify the fractal-like structure of the CAM microcirculation.

Our analysis refers only to 2D structures and needs to be extended to the more realistic case of 3D microvascular networks. It is important, however, to stress that important applications to 2D microcirculatory systems have already been investigated, e.g. by De Felice and colleagues [33]. In particular, they were able to show that D_f _could discriminate the oral vascular networks (gingival and vestibular oral mucosa) from controls and carriers of hereditary non-polyposis colorectal cancer (Lynch Cancer Family Syndrome II), i.e. D_f _is a marker for LCFS2.

Our general conclusions is that, if caution is paid in the selection of the images, in their handling, and in the selection of the code, the fractal dimension D_f _can represent a fast, reliable and robust parameter for evaluating angiogenetic processes. After further validation and refining, such parameters may potentially be useful in vascular and vascular-related diagnostics, for instance in monitoring the effectiveness of tumor anti-angiogenic therapies, anti-proliferative treatments in autoimmune syndromes and retinopathies and for stadiation of tumor developmental phases. Such applications would be an interesting extension to previous results, which have been considered to be relevant for diagnosis and therapy, i.e. that the fractal dimension is a prognostic factor for laryngeal carcinoma [34], for endometrial carcinoma [35] and for ovarian cancer [36]. Even more important, allometric scaling invariance has been observed in tumor growth processes involving lymph nodes [16], and could have a clinical impact in the future.
